# Multi-mode fault diagnosis datasets of gearbox under variable working conditions

**DOI:** 10.1016/j.dib.2024.110453

**Published:** 2024-04-18

**Authors:** Shijin Chen, Zeyi Liu, Xiao He, Dongliang Zou, Donghua Zhou

**Affiliations:** a MCC5 Group Shanghai Co. LTD, 201900, ShangHai, China; b Department of Automation, Tsinghua University, 100084, Beijing, China; c College of Electrical Engineering and Automation, Shandong University of Science and Technology, Qingdao 266590, China

**Keywords:** Gearbox, Variable working conditions, Fault diagnosis

## Abstract

The gearbox is a critical component of electromechanical systems. The occurrence of multiple faults can significantly impact system accuracy and service life. The vibration signal of the gearbox is an effective indicator of its operational status and fault information. However, gearboxes in real industrial settings often operate under variable working conditions, such as varying speeds and loads. It is a significant and challenging research area to complete the gearbox fault diagnosis procedure under varying operating conditions using vibration signals. This data article presents vibration datasets collected from a gearbox exhibiting various fault degrees of severity and fault types, operating under diverse speed and load conditions. These faults are manually implanted into the gears or bearings through precise machining processes, which include health, missing teeth, wear, pitting, root cracks, and broken teeth. Several kinds of actual compound faults are also encompassed. The development of these datasets facilitates testing the effectiveness and reliability of newly developed fault diagnosis methods.

Specifications Table

**Table:** 

Subject	Mechanical engineering
Specific subject area	Vibration, machine condition monitoring, fault diagnosis
Type of data	Datasets in “.csv” format
Data collection	Two three-axis acceleration sensors were used to collect the three-axis vibration acceleration signals of the motor drive end and the gearbox intermediate shaft respectively. The photoelectric sensor was used to collect the key phase signal (speed data) of the motor output shaft. The torque sensor was used to collect the torque data of the gearbox input shaft.
Data source location	MCC5 Group Shanghai Co. LTD Department of Automation, Tsinghua University
Data accessibility	Repository name: Multi-mode Fault Diagnosis Datasets of Gearbox Under Variable Working Conditions Data identification number: 10.17632/p92gj2732w.1 Direct URL to data: https://data.mendeley.com/datasets/p92gj2732w/1 Instructions for accessing these data:
Related research article	Z. Liu, C. Li and X. He, Evidential ensemble preference-guided learning approach for real-time multimode fault diagnosis, IEEE Transactions on Industrial Informatics, doi: 10.1109/TII.2023.3332112.

## 1 Value of the Data

• The data is collected from gearboxes operating under variable working conditions, including time-varying speed and load. The dataset includes vibration signals, speed signals, and torque signals, and encompasses a variety of fault types, including multiple single gear faults and multiple bearing-gear compound faults, as well as different degrees of severity. As depicted in Table 1, our dataset offers advantages over existing representative datasets.

**Table 1:** Comparison of several representative datasets.

	BJTU WT-planetary gearbox dataset [1]	SEU planetary gearbox dataset [2]	UO variable speed bearing dataset [3]	MCC5-THU gearbox fault diagnosis datasets
Number of fault types	5	5	3	7
Number of vibration signals	4	8	6	6
Sampling frequency	48 kHz	5.12 kHz	200 kHz	12.8 kHz
Sampling period	5 min	—	10 s	60 s
Number of steady conditions	8	2	0	24
Key variables	Speed	Speed, Load	Speed	Speed, Load
Number of transitional conditions	—	—	18	48
Number of compound faults	—	—	—	2
Number of fault degrees of severity	—	—	—	3
• Our dataset distinguishes itself from existing literature by incorporating data from more complex variable working conditions, a broader range of fault types and severity degrees, and multiple signals.
• This dataset enables the study of the time and frequency characteristics of gearbox fault signals under variable working conditions.
• The dataset can be utilized to evaluate the effectiveness of newly developed methods for gearbox fault diagnosis or condition monitoring under variable working conditions.

## 2 Background

The operating environment of gearboxes is both complex and harsh [4,5]. During the start-stop phase, the load, speed, lubrication conditions, and other operational parameters undergo changes, resulting in the gearbox functioning under time-varying speed and load conditions. Consequently, the variable working conditions (i.e., operating mode) of gearboxes lead to varying distributions of characteristics and frequencies for the same gear under different operating conditions. This variability, in turn, affects the robustness and accuracy of fault diagnosis models [6]. The significance of fault diagnosis algorithms lies in their capacity to efficiently identify and localize anomalies within complex systems, thereby facilitating timely and accurate remedial actions to ensure system reliability and performance optimization [7,8]. Therefore, it is of great significance to provide sufficient data support for studying fault diagnosis models under variable operating conditions.

## 3 Data Description

**Fig. 1:**
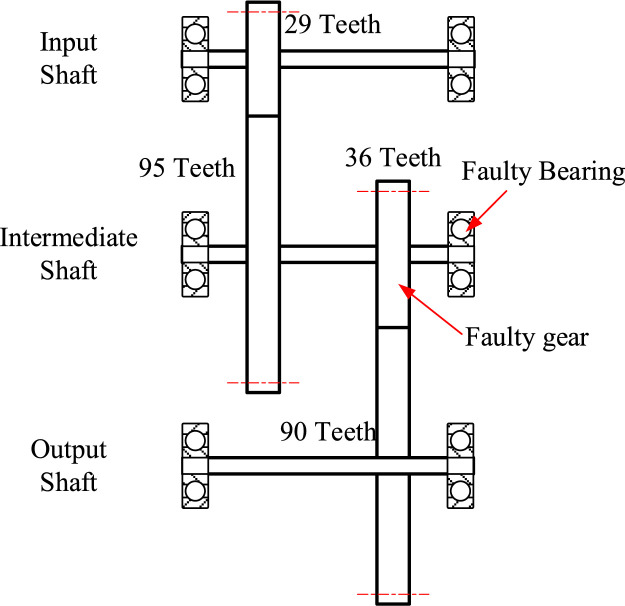
The internal structure diagram of the gearbox.

The datasets are collected based on different fault types, fault degrees, and working conditions, and contain a total of 240 sets of time series data. Each dataset contains 8 columns of data. The letters in the first row of each column indicate the meaning of the data in that column, as shown in Table 2. These datasets were collected based on a two-stage parallel gearbox under healthy and fault states. The internal structure diagram of the gearbox is shown in Fig. 1. The data can be employed to evaluate the effectiveness of methods developed for gearbox fault diagnosis under time-varying speed or load conditions, such as the methods proposed in the literature [9], [10], [11], [12].

**Table 2:** The meaning of each column in the dataset.

Letters	Meaning	Unit
speed	Motor output shaft key phase signal	dimensionless
torque	Gearbox input shaft torque	Nm
motor_vibration_x	Axial vibration acceleration of motor drive end	g
motor_vibration_y	Horizontal vibration acceleration of motor drive end	g
motor_vibration_z	Vertical vibration acceleration of motor drive end	g
gearbox_vibration_x	Axial vibration acceleration of gearbox intermediate shaft bearing seat	g
gearbox_vibration_y	Horizontal vibration acceleration of gearbox intermediate shaft bearing seat	g
gearbox_vibration_z	Vertical vibration acceleration of gearbox intermediate shaft bearing seat	g

Furthermore, the gear module is 1.5, and the gear width is 10 mm. The 36-tooth gear on the intermediate shaft is a faulty gear, and the support bearing model ER16K at the end of the intermediate shaft near the 36-tooth gear is a faulty bearing. The specific parameters of the utilized ER16K bearing are reported in Table 3.

**Table 3:** The specific parameters of utilized ER16K bearing.

Inner diameter	1 inch
Outer diameter	2.0472 inch
Width	0.749 inch
Ball diameter	0.3125 inch
Number of balls	9
Pitch diameter	1.516 inch

**Fig. 2:**
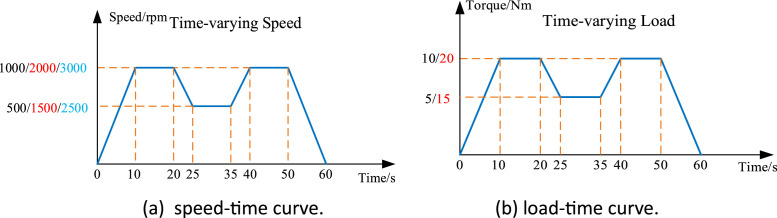
The set time-varying curves.

Each dataset was measured with a sampling frequency of 12.8 kHz. The datasets were stored in the standard Excel format, ".csv," in a single column without a time stamp. They were collected at time-varying speeds or time-varying loads for a fixed duration of 60 s, with the set speed-time curve and load-time curve depicted in Fig. 2(a) and (b), respectively. The number of colors in the figure is used to distinguish groups of experiments. Taking the 0-2500-3000 situation marked in blue in Fig. 2(a) as an example, it means that the speed is set to 3000 rpm within 10–20 s and within 40–50 s. At the same time, the rotation speed is set to 2500 rpm within 25–30 s.

During experiments under time-varying load conditions, the rotation speed was set to three constant values: 1000 rpm, 2000 rpm, and 3000 rpm. Similarly, during experiments under time-varying speed conditions, the load is set to two constant values: 10 Nm and 20 Nm.

The faults set in the experiment include both single gear faults and coincident gear and bearing faults. All faults were obtained by laser etching with a machining accuracy of 0.01 mm. The details corresponding to the fault parameters for bearings and gears are reported in Table 4, Table 5, respectively. In each scenario, in addition to the health status and "missing teeth" fault, three different fault degrees of severity are considered for the remaining four single faults ("gear wear," "gear pitting," "teeth crack," "teeth break") and two compound faults ("teeth break and bearing inner" and "teeth break and bearing outer"). Therefore, a total of 240 ".csv" files are included. In this context, a total of 24 steady conditions and 112 transitional condition combinations are considered.

**Table 4:** The details of bearing fault parameters.

Fault Parameter of Bearing	Fault Degree		
	Light	Medium	High
Inner Raceway Fault Width	0.1 mm	0.3 mm	0.5 mm
Outer Raceway Fault Width	0.1 mm	0.3 mm	0.5 mm

**Table 5:** The details of gear fault parameters.

Fault Parameter of Gear	Fault Degree		
	Light	Medium	High
Teeth Crack Depth	1/4 of the teeth height	1/2 of the teeth height	3/4 of the teeth height
Gear Wear	1/3 of the teeth surface area	1/2 of the teeth surface area	Full teeth surface area
Teeth Break	1/4 of the teeth width	1/2 of the teeth width	3/4 of the teeth width
Gear Pitting	Fault diameter 0.5 mm	Fault diameter 1.0 mm	Fault diameter 1.5 mm

Of note, the specific usage of the datasets is basically consistent with the usage of representative datasets. For researchers intending to leverage this dataset, we suggest constructing data encompassing the entire simulation lifecycle, reflecting real-world conditions, to evaluate the efficacy of their proposed methods. Additionally, researchers have the flexibility to generate tailored training and testing datasets as per their specific task demands and the contents of the provided .csv files.

**Fig. 3:**
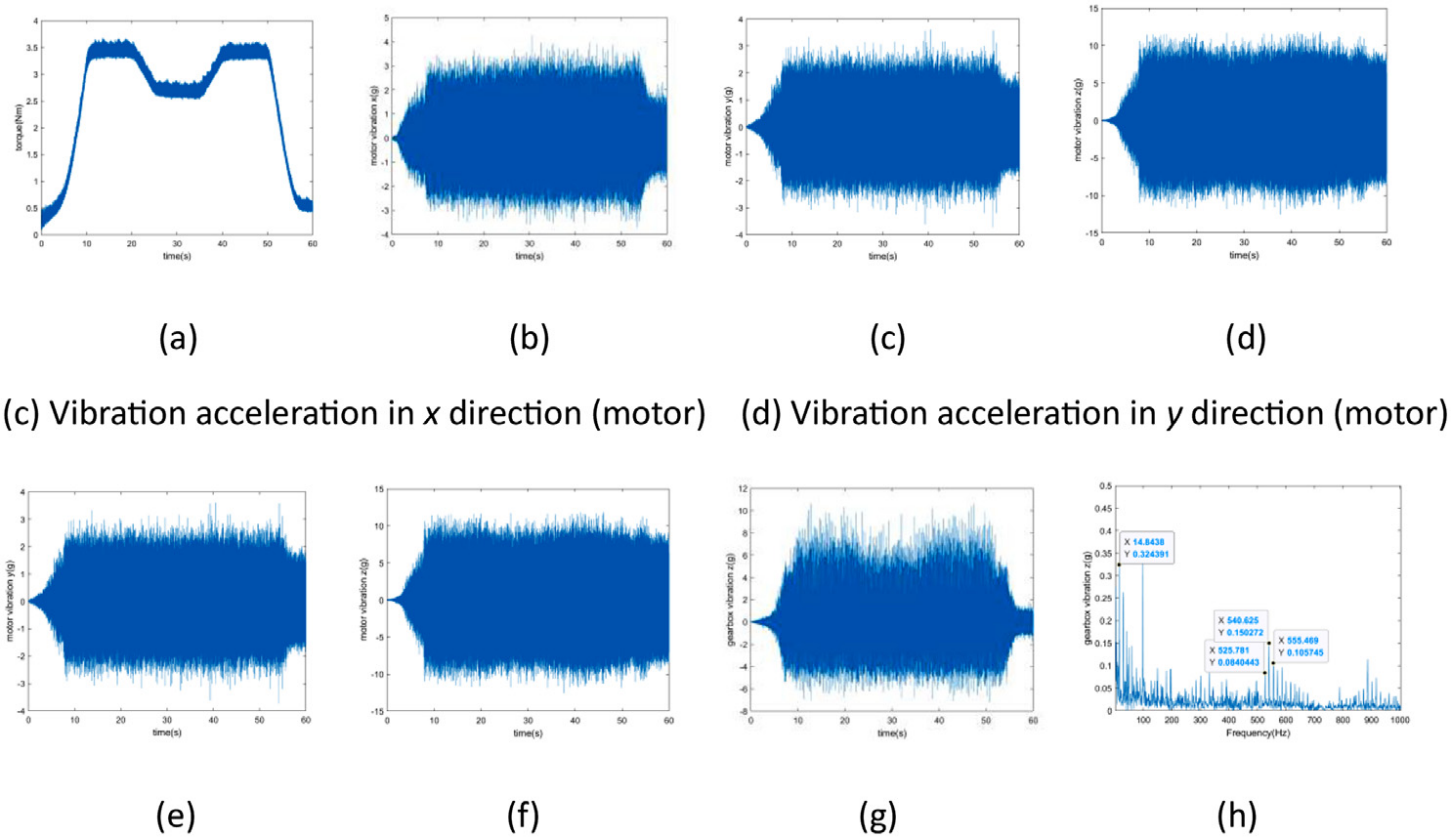
The visualization diagram of “miss_teeth_torque_circulation_3000rpm_20Nm” dataset. (a) represents the actual measured input shaft torque; (b-d) depict the vibration acceleration in the xyz directions of the motor; (e-g) showcase the vibration acceleration in the xyz directions of the gearbox's intermediate shaft; and (h) presents the envelope spectrum of the z-axis vibration acceleration of the gearbox's intermediate shaft.

Using the “miss_teeth_torque_circulation_3000rpm_20Nm” dataset as a case study, Fig. 3 presents the visualization diagram. It is noteworthy that the rotational frequency of the intermediate shaft is determined to be 14.84 Hz, while the meshing frequency of the small gear on the intermediate shaft is observed at 540.63 Hz, accompanied by its sideband frequencies at 525.78 Hz and 555.47 Hz. These observations align with the typical characteristics associated with missing teeth faults.

In addition, it should be noted that due to the hysteresis of the motor and torque generator, the actual speed-time curve or load-time curve may differ slightly from the set curve. The torque load of the gearbox output shaft is applied by the magnetic powder brake, while the torque borne by the gearbox input shaft is measured by the torque sensor. In this context, the actual signal has been measured by the key phase sensor and torque sensor.

To better understand the setting conditions corresponding to the dataset files, the meanings of some file names are explained as Table 6.

**Table 6:** Examples of the dataset file descriptions.

Filename	Description
gear_pitting_H_speed_circulation_10Nm_1000rpm	a gearbox dataset for high teeth surface pitting. Single pit diameter is 1.5 mm. Gear output shaft torque is 10 Nm. The motor input shaft rotates at the 0–1000 rpm speed-time curve shown in Fig. 2a.
gear_pitting_L_speed_circulation_20Nm_3000rpm	a gearbox dataset for light teeth surface pitting. Single pit diameter is 0.5 mm. Gear output shaft torque is 20 Nm. The motor input shaft rotates at the 0–3000 rpm speed-time curve shown in Fig. 2a.
gear_pitting_H_torque_circulation-1000rpm_10Nm	a gearbox dataset for high teeth surface pitting. Single pit diameter is 1.5 mm. The motor input shaft rotates at a constant speed of 1000 rpm. The torque of the gearbox output shaft is between 0–10 Nm, and the load-time curve is shown in Fig. 2b.
gear_pitting_M_torque_circulation_3000rpm_20Nm	a gearbox dataset for medium teeth surface pitting. Single pit diameter is 0.5 mm. The motor input shaft rotates at a constant speed of 3000 rpm. The torque of the gearbox output shaft is between 0–20 Nm, and the load-time curve is shown in Fig. 2b.
health_speed_circulation_20Nm-3000rpm	a gearbox dataset for health gearbox. Gearbox output shaft torque is 20 Nm. The motor input shaft rotates at the 0-3000rpm, and the speed-time curve is shown in Fig. 2a.
miss_teeth_torque_circulation_1000rpm_10Nm	a gearbox dataset for missing a teeth gearbox. The motor input shaft rotates at a constant speed of 1000 rpm. The torque of the gearbox output shaft is between 0–10 Nm, and the load-time curve is shown in Fig. 2b.
teeth_break_H_torque_circulation_1000rpm_10Nm	a gearbox dataset for high teeth break. Three quarters of a tooth is broken. The motor input shaft rotates at a constant speed of 1000 rpm. The torque of the gearbox output shaft is between 0–10 Nm, and the load-time curve is shown in Fig. 2b.
gear_wear_M_torque_circulation_1000rpm_10Nm	a gearbox dataset for medium gear wear. 1/2 of the tooth surface is worn. The motor input shaft rotates at a constant speed of 1000 rpm. The torque of the gearbox output shaft is between 0–10 Nm, and the load-time curve is shown in Fig. 2b.
teeth_crack_L_torque_circulation_1000rpm_10Nm	a gearbox dataset for light teeth crack. Teeth crack depth reaches 1/3 of tooth height. The motor input shaft rotates at a constant speed of 1000 rpm. The torque of the gearbox output shaft is between 0–10 Nm, and the load-time curve is shown in Fig. 2b.
teeth_break_and_bearing_inner_H_torque_circulation_1000rpm_10Nm	a gearbox dataset for high tooth break and bearing inner ring compound failure. Three quarters of a tooth is broken. The fault width of the inner raceway is 0.5 mm. The motor input shaft rotates at a constant speed of 1000 rpm. The torque of the gearbox output shaft is between 0–10 Nm, and the load-time curve is shown in Fig. 2b.
teeth_break_and_bearing_outer_H_torque_circulation_1000rpm_10Nm	a gearbox dataset for high tooth break and bearing outer ring compound failure. Three quarters of a tooth is broken. The fault width of the outer raceway is 0.5 mm. The motor input shaft rotates at a constant speed of 1000 rpm. The torque of the gearbox output shaft is between 0–10 Nm, and the load-time curve is shown in Fig. 2b.

## 4 Experimental Design, Materials and Methods

**Fig. 4:**
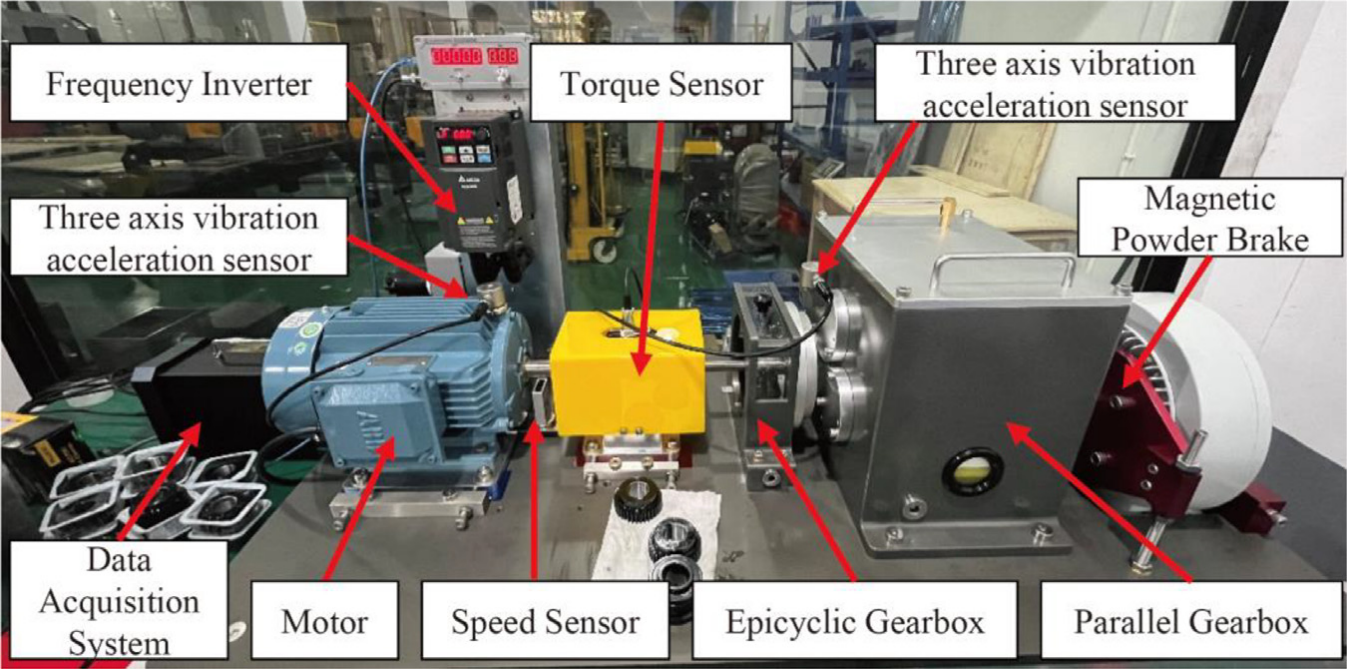
The actual Gearbox test rig.

The experimental setup for the dataset is shown in Fig. 4, which consists of a 2.2 kW three-phase asynchronous motor, a torque sensor, a two-stage parallel gearbox, a magnetic powder brake acting as a torque generator, and a measurement and control system. This dataset aims to simulate and document various fault conditions of the 36-tooth gear on the intermediate shaft and its adjacent support bearings under different operating modes. The magnetic powder brake is used to apply a torque load to the gearbox. The actual torque endured by the gearbox input shaft can be measured by a torque sensor (model S2001, synthetic accuracy: ±0.5 %F.S). The speed sensor is used to measure the key phase signal of the motor output shaft, and the motor output shaft speed can be calculated from the key phase signal. The test rig, as depicted in Fig. 4, is equipped with two three-axis vibration acceleration sensors (model TES001V, sensitivity: 100 mv/g) were used to measure motor and gearbox intermediate shaft triaxial vibrations along the x-, y-, and z-axes at a sampling frequency of 12.8 kHz. The datasets were collected and processed under 12 working conditions. In order to reduce the experimental error and measurement error caused by temperature, the temperature difference in the laboratory is controlled within the range of 2 ℃.

## Limitations

It is important to mention that this paper includes a wide range of experiments covering different working conditions, types of faults, and levels of severity. However, due to limitations in our experimental setup, we haven't conducted experiments at very high speeds, such as 30000 rpm. We plan to address this in future versions of our research.

## Ethics Statement

The authors have read and follow the ethical requirements for publication in Data in Brief and confirming that the current work does not involve human subjects, animal experiments, or any data collected from social media platforms.

## CRediT authorship contribution statement

**Shijin Chen:** Writing – original draft. **Zeyi Liu:** Conceptualization, Methodology, Writing – review & editing. **Xiao He:** Conceptualization, Supervision. **Dongliang Zou:** Conceptualization, Supervision. **Donghua Zhou:** Conceptualization, Supervision.

## Data Availability

Multi-mode Fault Diagnosis Datasets of Gearbox Under Variable Working Conditions (Original data) (Mendeley Data).
